# Incidence of reactive hyperplastic lesions in the oral cavity: a 10 year retrospective study in Santa Catarina, Brazil^☆^

**DOI:** 10.1016/j.bjorl.2018.03.006

**Published:** 2018-04-17

**Authors:** Kamile Leonardi Dutra, Lunardo Longo, Liliane Janete Grando, Elena Riet Correa Rivero

**Affiliations:** a Universidade Federal de Santa Catarina (UFSC), Programa de Pós-Graduação em Odontologia, Florianópolis, SC, Brazil; b Universidade Federal de Santa Catarina (UFSC), Faculdade de Odontologia, Florianópolis, SC, Brazil; c Universidade Federal de Santa Catarina (UFSC), Departamento de Patologia, Florianópolis, SC, Brazil

**Keywords:** Giant cell granuloma, Hyperplasia, Oral cavity, Pyogenic granuloma, Reactive hyperplastic lesions, Granuloma de células gigantes, Hiperplasia, Cavidade oral, Granuloma piogênico, Lesões hiperplásicas reativas

## Abstract

**Introduction:**

Reactive hyperplastic lesions develop in response to a chronic injury simulating an exuberant tissue repair response. They represent some of the most common oral lesions including inflammatory fibrous hyperplasia, oral pyogenic granuloma, giant cell fibroma, peripheral ossifying fibroma, and peripheral giant cell lesions.

**Objective:**

The incidence of those lesions was investigated in an oral pathology service, and the clinical characteristics, associated etiological factors, concordance between the clinical and histopathological diagnostic was determined.

**Methods:**

A total of 2400 patient records were screened from 2006 to 2016. Clinical features were recorded from biopsy reports and patients’ files.

**Results:**

A total of 534 cases of reactive hyperplastic lesions were retrieved and retrospectively studied, representing 22.25% of all diagnoses. The most frequent lesion was inflammatory fibrous hyperplasia (72.09%), followed by oral pyogenic granuloma (11.79%), giant cell fibroma (7.30%), peripheral ossifying fibroma (5.24%), and peripheral giant cell lesions (3.55%). Females were predominantly affected (74.19%), the gingiva and alveolar ridge were the predominant anatomical site (32.89%), and chronic traumatism was presented as the main etiological factor. The age widely ranges from the 1st decade of life to the 7th. Clinically, the reactive hyperplastic lesions consisted of small lesions (0.5–2 cm) and shared a strong likeness in color to the oral mucosa. The concordance between the clinical and histopathological diagnostic was high (82.5%).

**Conclusion:**

Reactive hyperplastic lesions had a high incidence among oral pathologies. The understanding of their clinical features helps to achieve a clearer clinical and etiological diagnosis, and the knowledge of factors related to their development. This may contribute to adequate treatment and positive prognosis.

## Resumo

**Introdução:**

As lesões hiperplásicas reativas se desenvolvem em resposta a uma lesão crônica que estimula uma resposta acentuada de reparo tecidual. Elas representam uma das lesões orais mais comuns, inclusive a hiperplasia fibrosa inflamatória, granuloma piogênico oral, fibroma de células gigantes, fibroma periférico ossificante e lesão periférica de células gigantes.

**Objetivo:**

A incidência dessas lesões foi investigada em um serviço de patologia bucal e as características clínicas, os fatores etiológicos associados e a concordância entre os diagnósticos clínico e histopatológico foram determinados.

**Método:**

Foram selecionados 2.400 registros de pacientes entre 2006 e 2016. As características clínicas foram registradas a partir de laudos de biópsia e dos prontuários dos pacientes.

**Resultados:**

Um total de 534 casos de lesões hiperplásicas reativas foram recuperados e retrospectivamente estudados, representando 22,25% de todos os diagnósticos. A lesão mais frequente foi hiperplasia fibrosa inflamatória (72,09%), seguida por granuloma piogênico oral (11,79%), fibroma de células gigantes, (7,30%), fibroma periférico ossificante (5,24%) e lesão periférica de células gigantes (3,55%). O sexo feminino foi predominante (74,19%), a gengiva e a crista alveolar foram o local anatômico predominante (32,89%) e o traumatismo crônico foi demonstrado como o principal fator etiológico. A idade variou desde a 1ª década de vida até a 7ª. Clinicamente, as LHR consistiram em pequenas lesões (0,5 a 2 cm) que apresentaram uma forte semelhança de cor com a mucosa oral. A concordância entre o diagnóstico clínico e histopatológico foi alta (82,5%).

**Conclusão:**

As lesões hiperplásicas reativas apresentaram alta incidência entre as patologias bucais. A compreensão das características clínicas ajuda na realização de um diagnóstico clínico e etiológico mais claro, bem como determinar os fatores relacionados ao seu desenvolvimento. Dessa forma contribui para um tratamento adequado e um prognóstico positivo.

## Introduction

Reactive hyperplastic lesions (RHL) of the oral cavity may develop due to a low-intensity chronic irritation that stimulates an exuberant tissue repair response. This exuberant response produces a soft tissue enlargement similar to a diverse group of pathologic processes. Consequently, this response represents a diagnostic challenge as an enlargement can be characteristic of a variation of normal anatomic structures, inflammation, cysts, developmental anomalies, and neoplasm.^1^

The RHL group is composed by Inflammatory Fibrous Hyperplasia (IFH), Oral Pyogenic Granuloma (OPG), Giant Cell Fibroma (GCF), Peripheral Ossifying Fibroma (POF), and Peripheral Giant Cell Lesions (PGCL). All of them share a likeness in similar clinical appearance to oral mucosa and represent the most common oral lesions, excluding caries, periodontal disease, and periapical inflammatory lesions.^1^

The clinical appearance of RHL is characterized by tissue growth, with fibrous or flaccid consistency, reddish color, sessile or pedunculated, and can occur in multiple intraoral sites. Usually, the gingiva is the most affected region for being exposed to irritation from the biofilm, calculus, food impaction, improperly adapted restorations or prostheses, and iatrogenic factors. Patients may report the absence of symptoms; or symptoms ranging from mild pain to bleeding. Radiographic findings are commonly absent, however, in rare cases of large lesions, a localized alveolar bone resorption could be noticed.^2^ Differential diagnosis includes hemangioma, Kaposi's sarcoma, bacillary angiomatosis, angiosarcoma, and Non-Hodgkin's lymphoma.^3^

Histopathologic examinations of the surgical specimens from oral biopsies are required in order to confirm the clinical diagnosis, and furthermore, to provide a definitive pathological diagnosis. This is done with the intention of an appropriate treatment being established to avoid the recurrence of the lesions. In this way, the aim of this study was to investigate the incidence of IFH, OPG, GCF, POF, and PGCL diagnosed by one reference oral-anatomopathological diagnostic service, as well as analyze the related demographic data and the correlation among clinical and histopathological diagnoses.

## Methods

This retrospective study was approved by the Ethics Committee for Research with Human Beings at the Federal University of Santa Catarina under number 1.097.375. All RHL cases were retrieved from the records of a Oral Pathology Laboratory, from 2006 to 2016. Cases in which the histopathologic diagnoses were IFH, OPG, GCF, POF, or PGCL were considered. Profile information was gathered on each case from biopsy files and patient records, and the following parameters were collected: patient's gender, age and ethnic group; and lesion-related data such as size, color, location and etiology. The cases were gathered and recorded consecutively so as to avoid any bias of selection. Additionally, the agreement among histopathological and clinical diagnoses issued by the dentist was assessed. Finally, the data collected was filled in a Microsoft Excel^®^ (Microsoft Corporation, Redmond, USA) dataset and descriptive statistical analysis was performed with all collected data using Microsoft Excel^®^ software.

### Literature review

A review of the literature was carried out to retrieve studies on RHL with no restriction of publication year or language. The inclusion criteria were retrospective studies, and case series that included at least 100 cases of RHL. Exclusion criteria were studies with no available full texts. An electronic search was done in Latin American and Caribbean Health Sciences (LILACS) and PubMed (including MedLine) on April 2017 and updated on January 2018. The following combinations of keywords summarize the search: “reactive hyperplastic lesions” OR “reactive lesions” OR “reactive gingival lesions” OR “oral pyogenic granuloma” OR “peripheral ossifying fibroma” OR “giant cell fibroma” OR “peripheral giant cell granulomas” OR “inflammatory fibrous hyperplasia”. All references were managed, and the duplicated hits were removed by reference manager software (Endnote X7, Thompson Reuters, New York, NY). The selection of the studies was performed in two phases. In phase 1, titles/abstracts that met the eligibility criteria were selected. If a title/abstract provided insufficient information for a decision on inclusion/exclusion, the full text was obtained and assessed in phase 2. Those who met the eligibility criteria were also included.

## Results

**Figure 1:**
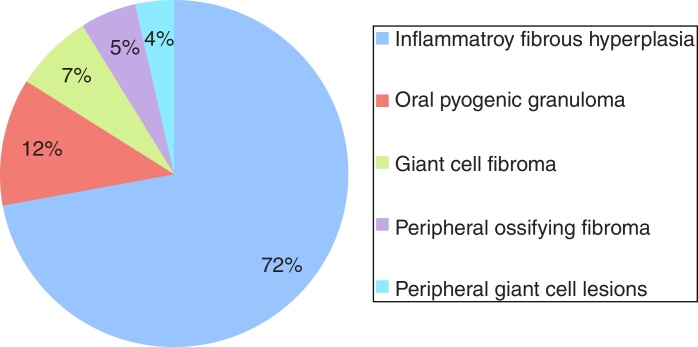
General incidence of lesions recorded as it relates to the histologic type.

**Figure 2:**
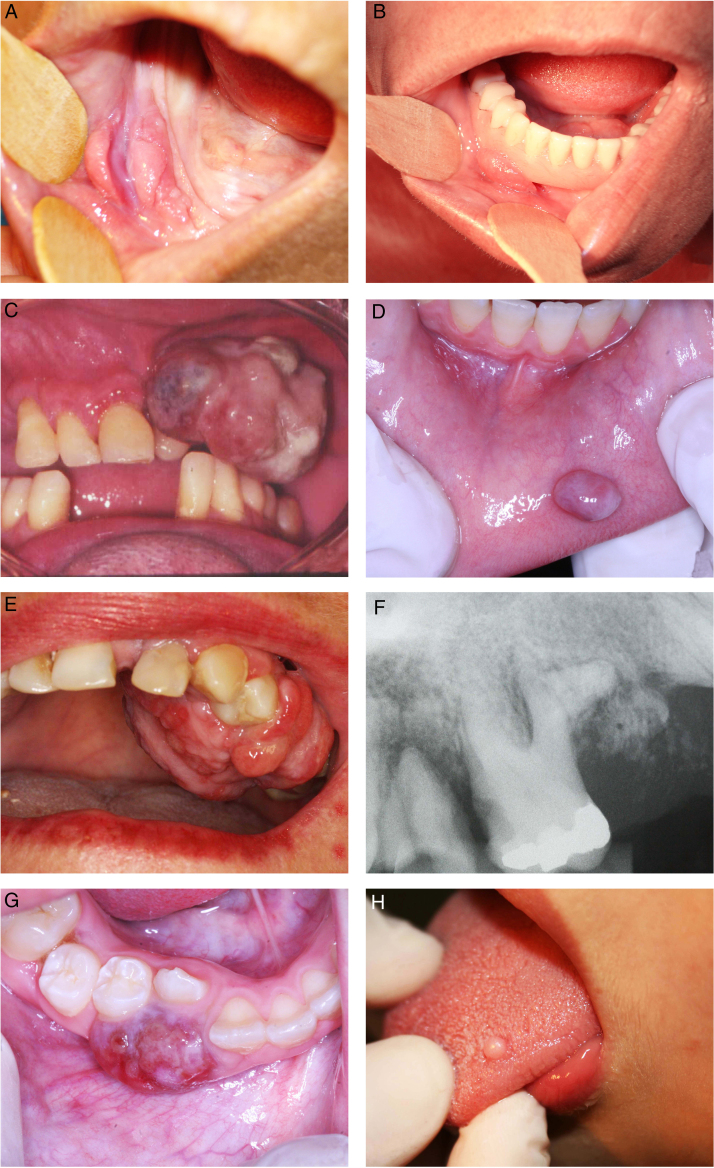
(A) Inflammatory fibrous hyperplasia; (B) ill-fitting denture over the inflammatory fibrous hyperplasia; (C) oral pyogenic granuloma in the alveolar ridge; (D) oral pyogenic granuloma in the lower lip; (E) peripheral ossifying fibroma; (F) periapical radiography of the peripheral ossifying fibroma; (G) peripheral giant cell lesions; (H) giant cell fibroma.

During the 10 years period, 2400 cases of oral lesions were biopsied and examined histologically at the Oral Pathology Laboratory. From this total, 534 cases were diagnosed as RHL, with an incidence of 22.25%. Fig. 1 illustrates the general incidence of lesions recorded as it relates to the histologic type. These cases occurred more frequently in females (74.19%), with the exception of PGCL which was the only group of lesions that demonstrated a higher incidence (61%) in males (Table 1). Amongst the women affected, 11.11% of the OPG cases were related to pregnant women. The patient age varied widely (Table 2). The preferential anatomic location was the alveolar ridge and jugal mucosa (Table 3). The recorded lesions were usually nodular, with the sizes ranging from 0.5 cm to 2 cm, resembling the color of the mucosa or slightly reddish-purple (Fig. 2). In all cases, the main etiological factor was ill-fitting dentures (Fig. 2B); excluding POF which presented the bacterial plaque as the main reported factor (Table 4).

**Table 1:** Cases diagnosed distributed by gender.

Diagnoses	Male		Female	
	*n*	%	*n*	%
Inflammatory fibrous hyperplasia	91	24	294	76
Oral pyogenic granuloma	16	26	46	74
Giant cell fibroma	15	38	24	62
Peripheral ossifying fibroma	04	14	24	86
Peripheral giant cell lesions	11	61	07	39

**Table 2:** Distribution according to the patients’ age.

Diagnoses	Age group (%)						
	0–19	20–29	30–39	40–49	50–59	60–69	<70
Inflammatory fibrous hyperplasia	03	02	10	22	32	22	10
Oral pyogenic granuloma	22	18	17	13	22	3	5
Giant cell fibroma	22	14	08	33	24	3	–
Peripheral ossifying fibroma	11	25	22	14	14	14	–
Peripheral giant cell lesions	45	11	5.5	11	11	11	5.5

**Table 3:** Distribution according to the predominant location of the lesion.

Diagnoses	Gingival/alveolar ridge	Tongue	Jugal mucosa	Palate	Lip	Other	*n*
Inflammatory fibrous hyperplasia	85	55	80	32	67	66	385
Oral pyogenic granuloma	34	13	01	02	11	02	63
Giant cell fibroma	10	19	06	02	01	–	38
Peripheral ossifying fibroma	28	–	–	–	–	–	28
Peripheral giant cell lesions	18	–	–	–	–	–	18

**Table 4:** Distribution according to the etiological factor.

Diagnoses	Ill-fitting denture	Chronic injury	Remaining root	Dental extraction	Bacterial plaque	Implants	Orthodontic brackets	Others	*n*
IFH	169	68	–	–	–	–	03	04	244
OPG	07	11	–	01	04	02	–	–	25
GCF	10	04	–	–	–	–	–	–	14
POF	01	01	01	01	03	–	–	–	07
PGCL	02	01	01	01	–	–	–	–	05

Total	189	85	02	03	07	02	03	04	295

IFH, inflammatory fibrous hyperplasia; OPG, oral pyogenic granuloma; GCF, giant cell fibroma; POF, peripheral ossifying fibroma; PGCL, peripheral giant cell lesions.

The most frequently observed lesion in this retrospective study was IFH, representing 72% of the diagnosed cases. IFH occurred mainly in females with a mean age of 50 years old, varying from 19 to 90 years, with a higher incidence evident during the 6th (32.4%), 5th (22.2%) and 7th (22.2%) decades of life. The preferential anatomic location was the alveolar ridge (22.0%) and jugal mucosa (20.7%). The lesion size varied from smaller than 0.5 cm (25.3%), 0.5–1.0 cm (30.14%), and 1.0–1.9 cm (22.58%). The most common corresponding etiological factors were ill-fitting dentures (68.6%), followed by chronic trauma (27.6%).

The remaining cases were OPG (12%) followed by GCF (7%), POF (5%), and PGCL (4%). Regarding OPG, females were mainly affected (74.2%) with a higher incidence noticed during the 1st (21.6%), 2nd (21.6%), 3rd (18.3%) and 6th (21.6%) decades of life. Within these cases, 11.6% were related to pregnant women, and 8.3% were recurrences. The preferential anatomic location was the alveolar ridge (53.9%) and the lips (20.6%). The lesion size varied from smaller than 0.5 cm (14.7%), 0.5–1.0 cm (32.3%), and 1.0–1.9 cm (38.2%). The greatest corresponding etiological factor was chronic trauma (44%) followed by ill-fitting dentures (28%).

GCF occurred mainly in females (61.5%) with a higher incidence noticed during the 4th (32.4%), 5th (24.3%), 1st (21.6%) and 2nd (21.6%) decades of life. The preferential anatomic location was the tongue (50%). The predominant lesion size was smaller than 0.5 cm (51.7%). The greatest corresponding etiological factor was ill-fitting dentures (71.4%) followed by chronic trauma (28.5%).

POF mostly affected females (85.7%) during the 3rd (25.0%) and 4th (21.4%) decades of life. All lesions recorded were on the gingiva and alveolar ridge. The most common lesion size was 1.0–1.9 cm (29.4%). Bacterial plaque was the etiological factor reported more frequently (27.2%).

PGCL had a greater incidence in males (61.1%) during the first and second decades of life (45.0%). All of these cases occurred in the gingival and alveolar ridge with the most frequent size between 1 cm and 1.9 cm (33.3%). Ill-fitting dentures (40%) were the main etiological factor involved, followed by chronic trauma (20%).

**Figure 3:**
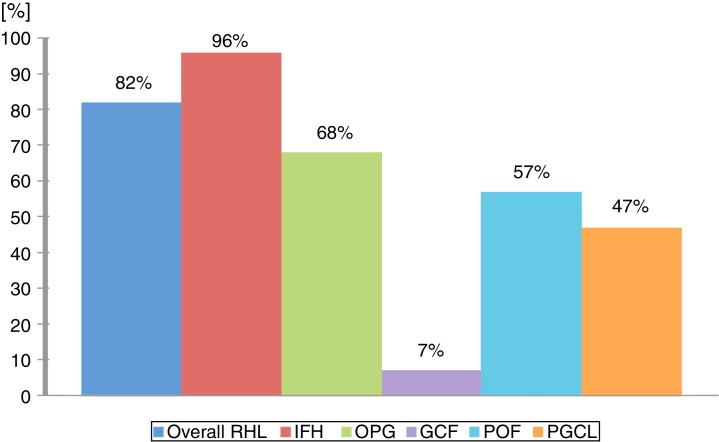
Distribution according to concordance among clinical and histopathological diagnoses.

**Figure 4:**
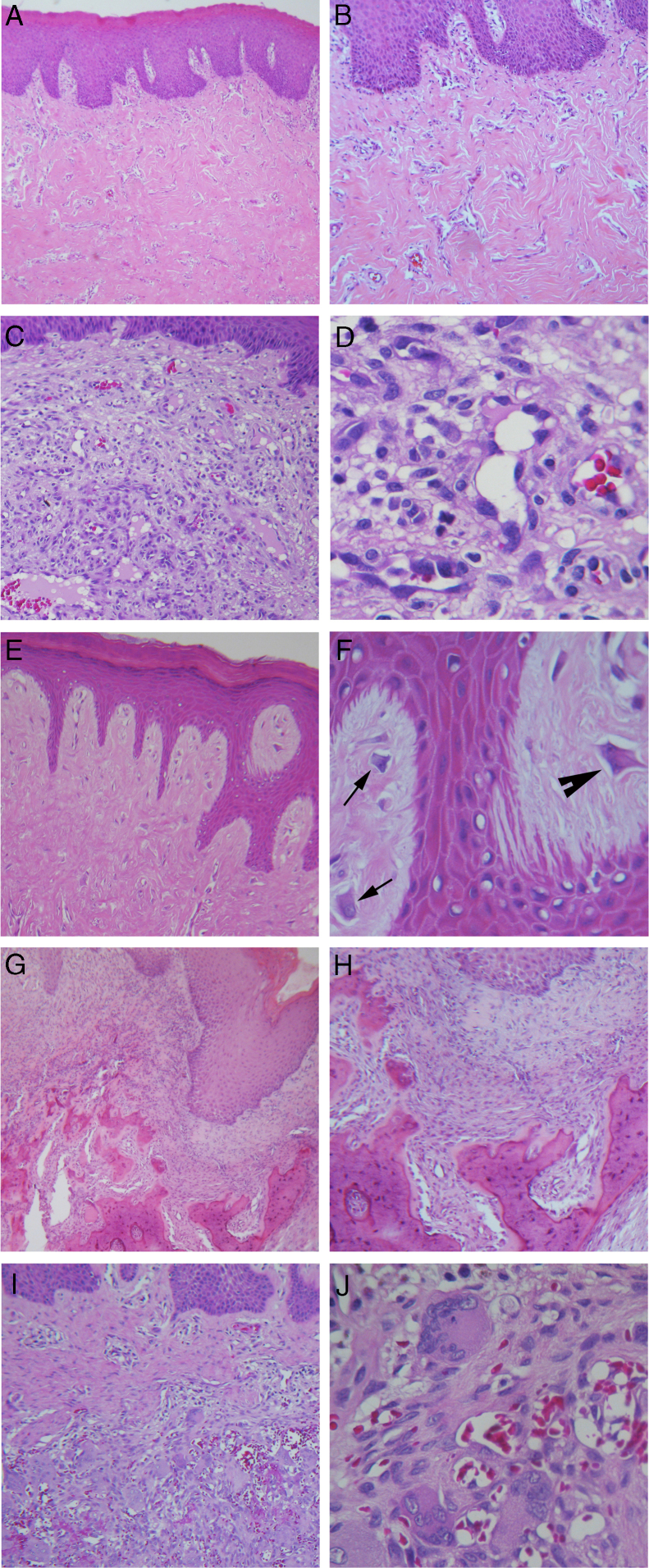
Hematoxylin and eosin staining. (A) Inflammatory fibrous hyperplasia (40×); (B) high magnification of the same case showing thick collagen fibers (100×); (C) oral pyogenic granuloma (100×); (D) high magnification of the same case showing greater number of endothelial cells and newly formed blood vessels (400×); (E) giant cell fibroma, with thin and long epithelial projections (100×); (F) high magnification of the same case showing the presence of stellate-shaped (arrow) and multinucleated (arrowhead) fibroblasts (400×); (G) peripheral ossifying fibroma, with mineralized product in the connective tissue (40×); (H) high magnification of the same case demonstrating irregular bone trabeculae formed (100×); (I) peripheral giant cell lesions (100×); (J) high magnification of the same case with large number of multinucleated giant cells associated with hemorrhagic areas (400×).

In general, the correlation among clinical and histopathological diagnoses was high; 370 out of 385 RHL's lesions received the same diagnosis from a clinical examination and a histopathologic exam. However, this concordance widely varied according to the lesion type; the higher concordance was observed in IFH lesions (96%), while GCF (7%) was lower (Fig. 3). Detailed information regarding histopathological features can be found in Fig. 4. And all detailed demographic data are reported in Table 1, Table 2, Table 3, Table 4.

The literature review search yielded 369 citations across electronic databases. Thereafter, inclusion and exclusion criteria were applied during a comprehensive evaluation of titles and abstracts, enabling the selection of 47 studies for full-text reading. Lastly, seven long-term studies that used a similar methodology of this present study were summarized in Table 5, comprising 12,229 oral lesions reviewed from biopsy files with a total of 2285 RHL, with an overall prevalence of 18.6%. These studies were conducted in different continents: Iran (123 cases),^4^, ^5^ Chile (1146 cases),^6^ India (773 cases),^7^, ^8^, ^9^ and Brazil (243 cases).^10^ Four articles showed that RHL most affected females patients in the third decade of life. Concerning localization, the alveolar ridge was the most frequent site followed by jugal mucosa and tongue; less frequently palate and lip were cited (Table 5). To date, no systematic review or meta-analysis covering this theme was identified.

**Table 5:** Long-term results of reactive hyperplasic lesions recorded from the literature. Data are percentage (%) unless otherwise stated.

Author, year, country	Total sample (*n*)	RHL	Histopathological diagnosis	Gender female	Age group (y) (mean age)	Predominant location					
						Alveolar ridge	Tongue	Jugal mucosa	Palate	Lip	Other
Zarei et al., 2007 – Iran	172	100.0^a^	IFH 19.1	26.0	14–76 (39.0)	–	–	–	–	–	–
			OPG 26.1	31.0	7–64 (27.0)	–	–	–	–	–	–
			POF 10.4	10.0	10–49 (24.0)	–	–	–	–	–	–
			PGCL 18.6	13.0	5–72 (13.0)	–	–	–	–	–	–

Amirchaghmaghi et al., 2011 – Iran	123	100.0^a^	IFH 40.6	36.0	(39.0)	20.0	28.0	30.0	8.0	10.0	4.0
			OPG 25.2	19.0		64.5	3.2	9.6	9.6	6.4	6.4
			POF 5.6	6.0		71.4	0.0	14.2	14.2	0.0	0.0
			PGCL 27.6	19.0		85.2	0.0	2.9	2.9	0.0	8.8

Maturana-Ramírez et al., 2011 – Chile	6369	18.0	IFH 71.1	70.7	0–19 (31.1)	18.5	19.4	20.6	0.0	16.6	24.7
			OPG 21.1	79.3	20–29 (8.5)						
			POF 2.9	57.1	30–39 (12.7)						
			PGCL 5.0	48.4	40–49 (19.5)						
					<50 (47.3)						

Kashyap et al., 2012 – India	240	41.6	IFH 35.0	31.0	16–59	34.2	14.2	51.4	0.0	0.0	0.0
			OPG 42.0	U	(34.0)	100.0	0.0	0.0	0.0	0.0	0.0
			POF 18.0	38.8	(39.0)	–	–	–	–	–	–
			PGCL 10.0	50.0	(33.0)	–	–	–	–	–	–

Reddy et al., 2012 India	1634	12.7	IFH 57.4	36.5	(74.1)	74.1	4.1	12.5	5.0	4.1	0
			OPG 18.7	74.3	(28.0)	89.7	0.0	5.1	0.0	5.1	0.0
			POF 17.7	72.9	(32.4)	100.0	0.0	0.0	0.0	0.0	0.0
			PGCL 6.22	53.8	(29.1)	100.0	0.0	0.0	0.0	0.0	0.0

Palmeira et al., 2013 – Brazil	938	35.0	IFH 82.6	73.0	40–59 (44.0)	40.9	14.0	16.1	12.2	11.0	5.8
			OPG 11.1								
			PGCL 6.1								

Vidyanath et al., 2015 – India	2753	10.7	IFH 51.9	71.4	–	64.8	9.5	18.6	3.1	3.4	0.6
			OPG 26.3								
			POF 9.8								
			PGCL 3.0								

U, unclear information reported; y, years; –, not reported information; IFH, inflammatory fibrous hyperplasia; OPG, oral pyogenic granuloma; GCF, giant cell fibroma; POF, peripheral ossifying fibroma; PGCL, peripheral giant cell lesions.

a These studies reported only RHL, thus the total sample is equal to the RHL sample.

## Discussion

Our findings revealed that RHL had a high incidence among oral pathologies. From the total of 2400 biopsy files and patient records surveyed, 534 concerned RHL (22.25%), which is inside the range found in the literature, from 10.7%^9^ to 41.6%.^7^ IFH was the most frequently encountered lesion in our study (72% of total RHL), and was also the most commonly biopsied lesion from the gingiva/alveolar ridge, jugal mucosa, lips, and tongue. The results are closest to Maturana-Ramírez et al. (71.1%)^6^ and Palmeira et al. (82.6%).^10^ However, Zarei et al.^4^ and Kashyap et al.^7^ reported OPG as the most frequent lesion, and IFH as the second with a prevalence of 19.1% and 35.0% respectively. The higher incidence of IFH was noticed during the fifth, sixth, and seventh decades of life in agreement with all studies reviewed, which probably is related to the use of an oral prosthesis. Previous studies attributed the development of IFH to a long-term use of a removable prosthesis or bad usage conditions, as it represents a constant injury to the oral tissues.^11^, ^12^, ^13^ Notably in the present study, more than two-thirds of the IFH cases had an ill-fitting prosthesis as their etiological factor.

OPG was the second most frequent lesion (12%) equally to Palmeira et al. (11.1%),^10^ Reddy et al. (18.7%),^8^ Maturana-Ramírez et al. (21.1%),^6^ and Vidyanath et al. (26.3%).^9^ This lesion represents a disorder of the oral mucosa, which usually appears as an inflammatory response with similar characteristics to those of a granuloma. That is the origin of the name, although not histologically representing a real granuloma.^3^ Consistent with other studies, OPG could be related to hormonal changes, specifically, with the vascular effects of female hormones. In this way, a higher incidence of OPG is expected in women, as this present study exhibited in accordance with Maturana-Ramírez et al.,^6^ Reddy et al.,^8^ and Palmeira et al.^10^ Moreover, this study revealed that 11.11% of OPG cases were related to pregnant women, which reinforces that theory. The predominant location of OPG was the gingiva/alveolar ridge, which is in agreement with the findings of Torrão et al.,^14^ Bertoja et al.,^12^ Amirchaghmaghi et al.,^5^ Kashyap et al.,^7^ Reddy et al.,^8^ Palmeira et al.,^10^ and Vidyanath et al.^9^ The common color among the lesions was reddish (69.23%), which could be explained by its typical histological constitution of a highly vascularized granulation tissue full of blood vessels. Regarding the correlation among clinical and histopathological diagnoses, a discordance of 31.7% was found similar to Tatli et al.^15^ and Vaz et al.^16^

GCF ranked third among RHL (7%), mostly consisting of lesions smaller than 0.5 cm. The studies clustered in Table 5, that reported RHL prevalence from a large pool of cases surveyed, did not cite GCF in their results. Thus, for this pathology, specific studies needed to be gathered in order to review its clinic-pathological features. This may be linked to the fact that GCF had shown a lower rate of concordance among clinical and histopathological diagnoses in our study, which may indicate that GCF is a lesion less familiar among clinicians. Clinically, GCF may resemble other fibrous growths like irritation fibroma and may often be diagnosed as IFH,^17^ which is the most common RHL and probably is the most remembered lesion by the clinicians. A greater incidence was also found among women in the fourth decade of life, thereby corroborating with Weathers and Callihan^18^ and Sabarinath et al.^19^ In our study, GCF differentiated from the other RHLs as the tongue served as the main site. A consensus about the etiology and pathogenesis of GCF remain unclear. The most accepted hypothesis for its origin is a response to trauma or to recurrent chronic inflammation,^20^ characterized by functional changes in fibroblastic cells, while other cells would take over for collagen synthesis.^21^ In contrast, some authors defined GCF as a benign neoplasm that is unaffiliated with traumatic factors.^22^ In this study, all cases of GCF were related to chronic trauma; mostly involved ill-fitting dentures as the etiological factor, and four of them were related to chronic injury with no specific reason recorded.

POF accounts for 5.2% of all RHL in this study, reported mainly in women in the third and fourth decades of life. The frequencies described in other studies range from 2.9% in Chile^6^ to 18.0% in India,^7^ also mainly noticed in women in the third and fourth decades of life. POF has been reported as a common solitary gingival overgrowth arising from the gingival, periosteum or periodontal ligament.^23^, ^24^ In this study, all POF lesions were restricted to gingival and alveolar ridge sites, equally to Reddy et al.^8^ However, POF lesions had already been found in palate and jugal mucosa.^5^ Probably the excessive proliferation of tissue resulted from gingival irritation by poor oral hygiene as a precipitating factor.^4^ Regarding the concordance among clinical and histopathological diagnoses, a moderate concordance of 57% was found.

PGCL was the only group of lesions that demonstrated a greater incidence amongst males, which corroborated with Kfir and Hansen,^25^ Zarei et al.,^4^ and Amirchaghmaghi et al.^5^ Other studies reported PGCL occurrence equally distributed by gender,^7^, ^8^ although a higher incidence amongst females had already been reported.^12^, ^26^ In the present study, the first and second decades of life had the higher incidence of cases, demonstrating a lesion more inclined to be found in younger people, which was not supported by the other studies reviewed. In our records, PGCL was characterized by multiple small reddish-purple (more than half of cases) to bluish nodules along the gingival sites only. This typically bluish-purple pigmentation is due to the presence of hemosiderin, which can be observed by the abundant hemorrhage throughout the lesion mass. Regarding the concordance among clinical and histopathological diagnoses, a concordance of 47.36% was found similar to Seifi et al.^27^

Most of the RHL represented an exuberant response to local irritation and trauma. Many different local irritants have been described: the present study identified ill-fitting dentures, chronic injury, remaining root, dental extraction, bacterial plaque, implants, and orthodontic brackets as potential etiological factors. In general, RHL were mostly nodular, with the size ranging from 0.5 cm to 2 cm, resembling the color of the mucosa or slightly reddish-purple, mainly affecting leukoderma women during the fifth and sixth decades of life. Although the greater number of mucosal biopsies among females may be related to a cultural bias,^11^ these findings were also found in other countries such as India^8^, ^9^ and Chile.^6^

## Conclusion

RHL had a high incidence among oral pathologies. Understanding their clinical features helps to provide a clearly definitive diagnosis as well as to identify the etiological factors related to their development, which contributes to insuring adequate treatment and positive prognosis without recurrence. The correlation among clinical and histopathological diagnoses was high for IFH lesions, moderate for OPG, POF, PGCL, and very low for GCF; which may reveal that clinicians were not familiar with RHL in general, and especially with GCF.

## Conflicts of interest

The authors declare no conflicts of interest.
